# Complex Roles of Microglial Cells in Ischemic Stroke Pathobiology: New Insights and Future Directions

**DOI:** 10.3390/ijms18030496

**Published:** 2017-02-25

**Authors:** Revathy Guruswamy, Ayman ElAli

**Affiliations:** 1 Neuroscience Axis, CHU de Québec Research Center (CHUL), Québec City, QC G1V 4G2, Canada; revathy.guruswamy.1@ulaval.ca; 2 Department of Psychiatry and Neuroscience, Faculty of Medicine, Laval University, Québec City, QC G1V 4G2, Canada

**Keywords:** ischemic stroke, injury, repair, microglia, macrophages, monocytes

## Abstract

Ischemic stroke constitutes the major cause of death and disability in the industrialized world. The interest in microglia arose from the evidence outlining the role of neuroinflammation in ischemic stroke pathobiology. Microglia constitute the powerhouse of innate immunity in the brain. Microglial cells are highly ramified, and use these ramifications as sentinels to detect changes in brain homeostasis. Once a danger signal is recognized, cells become activated and mount specialized responses that range from eliminating cell debris to secreting inflammatory signals and trophic factors. Originally, it was suggested that microglia play essentially a detrimental role in ischemic stroke. However, recent reports are providing evidence that the role of these cells is more complex than what was originally thought. Although these cells play detrimental role in the acute phase, they are required for tissue regeneration in the post-acute phases. This complex role of microglia in ischemic stroke pathobiology constitutes a major challenge for the development of efficient immunomodulatory therapies. This review aims at providing an overview regarding the role of resident microglia and peripherally recruited macrophages in ischemic pathobiology. Furthermore, the review will highlight future directions towards the development of novel fine-tuning immunomodulatory therapeutic interventions.

## 1. Introduction

Stroke is the most common cause of death and disability in the industrialized world. Ischemic stroke accounts for the majority of cases, whereas the remaining cases are hemorrhagic. Disruption of the regional blood supply initiates the ischemic cascade leading to neuronal dysfunction and subsequently death [1,2]. The ischemic cascade is governed by molecular events that trigger the breakdown of the blood–brain barrier (BBB) contributing to the secondary progression of injury by exacerbating brain edema and inflammation response in the sub-acute phase [1,3]. The severity of these early events reduces the capacity of neurons to recover in the chronic phase significantly worsening stroke outcomes [4,5]. Importantly, ischemic stroke results in two major zones of injury, the core or infarct that undergoes immediate cell death by necrosis, and the peri-infarct penumbra that undergoes delayed programmed cell death [6]. The slow progression of cell death within the penumbra implies that therapeutic salvage is possible. However, although significant progress has been made in stroke prevention and supportive care, still no disease-modifying therapy exists. Until now, recombinant tissue-plasminogen activator (rtPA)-induced thrombolysis remains the only food and drug administration (FDA) approved approach that is used in clinics to restore cerebral blood flow [6].

Upon injury, microglia, which are the resident macrophages of the brain, become activated and several circulating immune cells infiltrate the injured tissue [7,8]. Among these immune cells, monocytes, the precursors of tissue-infiltrating macrophages, play a particularly important role by giving rise to macrophages, which are morphologically similar to resident microglia [9,10]. In the acute phase, the inflammatory response appears to contribute to ischemic pathology, thereby anti-inflammatory strategies have been widely evaluated in experimental studies [11]. Unfortunately, attempts to translate these anti-inflammatory strategies into the clinics were very disappointing [12]. One proposed reason of this failure might be the dual role of inflammation in ischemic stroke pathobiology [12]. Indeed, emerging data are suggesting that microglial cells play complex and multiphasic roles after ischemic stroke displaying both adverse and beneficial effects [13,14]. As such, this review aims to summarize and discuss the recent findings addressing the spatiotemporal role of microglia in ischemic stroke pathobiology and the direct implications on therapies.

## 2. Origin and Physiological Roles of Microglia

Microglia are mononuclear phagocytes that constitute the main resident immune cell population of the brain, representing up to 10% of total brain’s cells [15]. Despite the extensive research conducted since their discovery in 1919, the origin of microglia is still elusive. Different possible origins have been proposed; some have proposed that microglia are derived from progenitors that originate from the neuroectoderm and/or the mesoderm colonizing the brain at the early embryonic stage throughout the fetal development stage [16,17,18], while others have proposed that microglia are derived from circulating blood monocytes during the late gestational stage throughout the early postnatal stage [19,20]. Recent fate-mapping studies showed that under physiological conditions microglia are not derived from the bone marrow but rather from myeloid stem cells in the yolk sac [21,22]. Nowadays, a consensus has been reached suggesting that microglia are derived from myeloid progenitors that infiltrate the brain during the different stages of brain development. Importantly, the majority of microglial cell population is generated during the post-natal stage after BBB formation [23,24]. A salient aspect of the brain microenvironment is the presence of the BBB that separates the blood circulation from the brain. For this reason, unlike any other tissue-resident macrophages, microglia evolve in a highly specialized microenvironment characterize by limited contacts and interactions with the blood-borne elements, unless under traumatic conditions [7]. Following brain infiltration, microglia evolve in a highly specialized microenvironment characterized by the presence of developing neurons and radial glia, but devoid of other glial cells [25,26]. Another question that is also debated is how microglial turnover is maintained in the adult brain throughout the lifespan. It is now widely accepted that microglia are maintained in the adult brain through either a self-renewal process or by the expansion of a reservoir of progenitors that have colonized the brain early during the embryonic stage [27,28].

Regardless their exact origin, once microglial progenitor cells have infiltrated the brain, they adopt a highly ramified phenotype with a small soma [29,30]. Originally, it was suggested that this ramified phenotype translates a resting state. However, recent findings demonstrated that microglial cells are never resting and are continuously patrolling the brain in order to maintain the integrity of brain tissue [26]. These findings showed that microglia use their motile ramifications as sentinels to survey and scan the microenvironment within their vicinity in order to detect any occurring change in brain homeostasis [26]. Once they detect a threat, microglia rapidly become activated by adopting amoeboid phenotype with a large soma [29]. Activated microglia mount adequate responses, which could range from elimination of cell debris by phagocytosis to the release of bioactive molecules that include immune and non-immune factors [31]. Several studies have revealed that under physiological conditions, microglia use their motile ramifications to establish specific and repeated cell contacts with neurons, astrocytes and endothelial cells [32,33,34]. Microglia are very dynamic and undergo extreme remodeling process throughout the lifespan, translating their complex role as regulators of neuronal function under physiological conditions [22,26,35]. In the healthy brain, microglia support neuronal function via two major mechanisms, phagocytosis, and biochemical interactions. Originally, it was thought that the phagocytic function of microglia is exclusively related to pathological conditions. However, recent data demonstrated that microglia are involved in the phagocytosis of cell debris that are generated by apoptotic cells in regions undergoing active neurogenesis in the adult brain, and in shaping circuit function [26,35,36]. Importantly, microglia constitute the main source of reactive oxygen species (ROS), which are involved in degrading the internalized targets inside phagosomes [37]. Interestingly, if these targets are not correctly processed inside phagosomes, this can lead to the release of toxic molecules by microglia in cell’s surrounding, including ROS, a phenomenon termed as “frustrated phagocytosis”, which usually occurs when the targets that are to be engulfed and internalized are too large [38,39]. The normal phagocytic process is accompanied by the release of several anti-inflammatory cytokines, growth and neurotrophic factors, and reduces the release of several pro-inflammatory cytokines [40]. Microglia has been shown to establish tight contacts with the synapses [26]. Through these contacts, microglia shape neuronal circuit function by eliminating synaptic elements (specifically, transient dendritic spines) via phagocytosis [26,35,41]. Furthermore, microglia are involved in maintaining the function of neurons and synaptic plasticity via complex biochemical interactions that include regulation of the neurotransmission and secretion of several proteolytic enzymes such as matrix metalloproteases and tissue-type plasminogen activator which are involved in remodeling the extracellular space within the synapses [26].

## 3. Microglial Cell Signaling

As discussed above, the dynamic nature of microglia is essentially due to their capacity to undergo extreme remodeling process throughout the lifespan. This characteristic requires a strict control over several cell functions that include regulation of the ramification movement, overall cell motility, cell morphology, phagocytosis, immune functions and the secretion of bioactive molecules. Several signaling pathways control these functions. A wide range of surface molecules controls the function of microglial cells. Importantly, over 100 genes expressed by microglia have been identified to be involved in sensing cell’s microenvironment [42]. This list includes several genes encoding for specialized transmembrane receptors or and clusters of differentiation (CD) markers, along with some secreted proteins [42]. Moreover, around 239 genes and eight microRNAs have been shown to be uniquely or highly expressed in microglia compared to myeloid and other immune cells [43]. Besides, around 29 genes distinguish microglia from other brain cells as well as from peripheral myeloid cells [43]. Here, we will briefly present major signaling pathways that play essential roles in controlling key functions of the microglia.

### 3.1. Pattern Recognition Receptor (PRR) Signaling

Microglia express several receptors involved in innate immunity, such as PRRs that regroup three major receptor families, toll-like receptors (TLRs), nucleotide-binding oligomerization domain (nod)-like receptors (NLRs), and the retinoic acid-inducible gene-1 (RIG1)-like receptors (RLRs). These receptors recognize specific ligands, or “patterns,” called pathogen-associated molecular patterns (PAMPs) and danger-associated molecular patterns (DAMPs). These patterns include proteins, such as exogenous peptidoglycans and endogenous heat shock proteins, and non-protein molecules, such as adenosine triphosphate (ATP) and nucleic acid molecules [44]. Activation of the PRRs induces specific signaling pathways that are involved in modulating microglial functions by transducing via two major intracellular mechanisms that converge towards nuclear factor-kappa B (NF-κB) signaling pathway, and toll/interleukin-1 receptor (TIR)-domain-containing adapter-inducing interferon-β (TRIF) that induces interferon regulatory factor-3 (IRF3) signaling pathway [45].

### 3.2. Cytokine Receptor Signaling

Microglia also express several cytokine receptors that directly contribute to modulation of the cell function. Additionally, they produce a large repertoire of cytokines that are essential for microglial cell function, among which are the tumor necrosis factor-α (TNFα), transforming growth factor-β (TGFβ), and interleukin signaling pathways play major roles [45]. Microglia express the TNF receptor 1 (TNFR1/p55) and TNF receptor 2 (TNFR2/p75), and constitute the major source of TNFα in the brain [46]. TNFR1 has a higher binding activity for the soluble TNFα compared to TNFR2, whereas the latter has a higher binding affinity to the membrane-anchored TNFα [47]. These receptors play a key role in regulating cell–cell communication and interaction [47]. In contrast to TNFα that elicits pro-inflammatory responses by microglial cells, TGFβ was shown to elicit opposite anti-inflammatory responses [45]. TGFβ is a multifunctional cytokine that initially binds TGFβ receptor type II (TGFRII) inducing recruitment of the TGFβ receptor type I (TGFRI) [45]. This cytokine is released as inactive complex, which becomes active through different processes [48]. TGFβ signaling pathway has been shown to contribute essentially to the anti-inflammatory response by reducing the production of pro-inflammatory cytokines, the production of nitric oxide (NO) as well as the release of ROS [45]. However, new findings reveled that this cytokine can also contribute to promote inflammation by inducing the stimulation of T-helper (Th)17 cells [49] Finally, microglia were reported to express a high number of interleukin receptors (ILRs), namely IL1R, IL5R, IL6R, IL8R, IL9R, IL10R, IL12R, IL13R, and IL15R. IL1β and IL6 play particularly important roles in the microglial cell function [45]. The cytokines can be divided into two groups: pro-inflammatory cytokines that include IL1β and IL6, and anti-inflammatory cytokines that include IL4 and IL10 [45].

### 3.3. Chemokine Receptor Signaling

Chemokines constitute a group of bioactive molecules that trigger chemotaxis in nearby responsive cells. Chemokines include a large family of molecules characterized by the presence of conserved cysteine residues in their N-terminal sequences [45,50,51]. Based on the spacing of their first two-cysteine residues, they are classified into four distinct subgroups, C chemokines (one N-terminal cysteine), CC chemokines (two adjacent N-terminal cysteines), CXC chemokines (one amino acid between the two N-terminal cysteines), and finally CX3C chemokines (three amino acids between the two N-terminal cysteines) [45,50]. In the brain, chemokines are essentially produced by neurons, astrocytes, microglia and endothelial cells. All chemokines mediate their effects following their release as soluble molecules that generate a chemotactic gradient stimulating the mobilization, invasion and migration of responsive cells [45,50]. An exception to this rule is the CX3C ligand 1 (CX3CL1; fractalkine) that mediates its effect as either a soluble molecule or a membrane-anchored molecule [50]. Depending on the nature of the stimuli, the chemokines are functionally divided into two groups, homeostatic, which are constitutively produced and contribute in basal cell migration, and inflammatory, which are induced once the inflammatory response is engaged and contribute to the inflammatory response [52]. Chemokines mediate their effects in microglial cells through a wide range of receptors that include CCL1 receptor CCR1 to 7 and CXCR1 to 5 [29]. Chemokine receptors are G protein-coupled receptors (GPCRs), which upon activation modulate different downstream signaling pathways leading to regulation of the calcium (Ca^2+^) homeostasis, activity of the Rho GTPases, and modulation of the mitogen activated protein kinases (MAPK) signaling pathway. CCL2/CCR2 and CX3CL1/CX3CR1 signaling pathways play particularly important roles in regulation of the microglial cell function. TLR engagement triggers the release of CCL2 by microglia, acting in an autocrine and paracrine manner [45]. In parallel, CCL2/CCR2 signaling pathway plays a central role in regulating the recruitment of peripheral leukocytes and their infiltration into the brain during inflammatory reactions [53]. On the other hand, CX3CL1 is essentially produced by neurons binding its receptor CX3CR1 that is specifically expressed in microglia [36]. CX3CL1/CX3CR1 signaling pathway is essential for neuronal–microglial communication and interaction, and plays an important role in regulating microglial migration, which is essential for surveying the brain throughout the lifespan [36]. In parallel, CX3CL1/CX3CR1 signaling represses the production of several pro-inflammatory cytokines by microglia; thereby pathway inactivation impairs microglia cell migration and interaction with neurons resulting in an exacerbated inflammatory response [54].

### 3.4. Neurotransmitter Receptor Signaling

Microglia also express different types of neurotransmitter receptors. These receptors control for instance the interaction and crosstalk between microglia and neurons [29]. Among these neurotransmitter receptors, purinoceptors, glutamate receptors, cholinergic receptors, adrenergic receptors, and dopamine receptors have been shown to play essential roles in modulating microglial cell activity, as reviewed in [29].

### 3.5. Triggering Receptor Expressed on Myeloid Cells-2 (TREM2) Receptor Signaling

Emerging evidence is outlining a key role of TREM2 receptor in controlling key aspects of microglial cell function [55,56]. TREM2 is a cell surface receptor belonging to the immunoglobulin (Ig) superfamily family that is encoded by a gene cluster linked to major histocompatibility complex (MHC) [55,57]. Depletion of the TREM2 in vivo severely impairs microglial cell morphology, migration and cell capacity to respond to environmental stimuli [58]. TREM2 seems to act as a sensor for charged lipids that are derived from the cell membrane of neurons and glial cells [58]. Additionally, TREM2 was shown to bind the heat shock protein-60 (Hsp60) enhancing the phagocytic capacity of microglial cells [59].

### 3.6. Phosphatidylserine (PS) Receptor Signaling

PS is a phospholipid normally sequestered in the inner leaflet of plasma membrane, and becomes exposed once cells are undergoing apoptosis [60]. The efficacious clearance of cell debris by specialized phagocytes, such as microglia, requires the recognition of PS by specialized receptors (PSRs) [61,62]. Microglia express several PSRs that directly bind PS, like brain-specific angiogenesis inhibitor-1 (BAI1), T-cell immunoglobulin mucin receptor 1 (TIM1), and TIM4 [61]. PSRs include as well several receptors that indirectly bind PS by using an intermediate molecule, namely c-mer proto-oncogene tyrosine kinase (MerTK) and vitronectin receptors (αvβ3 or αvβ5 integrins) [61].

### 3.7. Scavenger Receptor (SRs) Signaling

SRs comprise structurally diverse cell membrane receptors that contribute to several cell functions, such as the uptake of the negatively charged macromolecules, and modified low-density lipoprotein (LDL) [63]. Several classes of SRs are expressed in microglial cells playing key roles in regulating the innate immune response [64,65]. Among these are macrophage SR class AI (SR-AI), macrophage receptor with collagenous structure (MARCO), SR-B3 (CD36), macrosialin (CD68), and lectin-like oxidized low-density lipoprotein receptor-1 (LOX1) [45].

### 3.8. Other Receptor-Mediated Signaling

Fc receptors (FcRs) belong to the Ig superfamily that binds the constant domain (Fc) of Ig. They are subdivided into different subclasses based on their binding to specific isotype classes and subclasses of Ig: FcαR binds IgA, FcδR binds IgD, FcμR binds IgM, FcεR binds IgE, and FcγR binds IgG [45]. Microglia express all FcR subgroups [66]. The sialic acid-binding immunoglobulin-type lectin-3 (Siglec-3; CD33) is a type 1 transmembrane receptor that is essentially expressed on the surface of cells that belong to the myeloid lineage [67]. CD33 is expressed by microglia and its activation significantly reduces their phagocytic capacity [68]. Moreover, Siglec-E, a CD33-related Siglecs has been recently shown to recognize neural glycocalyx and to inhibit the phagocytosis of neural debris by microglia in vitro [69]. Microglia express other set of receptors that include complement receptors (CRs), macrophage colony-stimulating factor receptor (m-CSFR), Sigma-1 receptor (S1R), progesterone receptor membrane component-1 (PGRMC1), OX2 (CD200) cell membrane glycoprotein receptor (CD200R), and receptor for advanced glycation endproducts (RAGE) [45].

### 3.9. Microglial Cell Markers

Until now, there is no specific microglial cell marker that enables to distinguish these cells from other macrophages and various monocyte-derived macrophages (MDMs) [70,71]. Most experimental studies use pan-markers combined to morphological identification to investigate microglial cell activation. However, these approaches do not allow discrimination of resident microglia and other infiltrated macrophages. One of the most commonly used pan-marker is Ionized calcium binding adaptor molecule-1 (Iba1) that is widely used in immunohistochemical analysis [70]. Additionally, some other markers, such as CD11b, isolectin (IB4), and F4/80, are also commonly used in experimental studies. Several studies used the expression level of hematopoietic cell surface marker CD45 as a marker for microglia. However, microglia express low levels of CD45, whereas infiltrated-hematopoietic cells express high levels of CD45 [72]. Furthermore, the lysosomal protein CD68 and the MHCII cell surface receptor human leukocyte antigen-antigen D related (HLA-DR) are widely used as markers for activated microglia [70,73].

## 4. Microglial Cell Responses Following Ischemic Stroke

**Figure 1:**
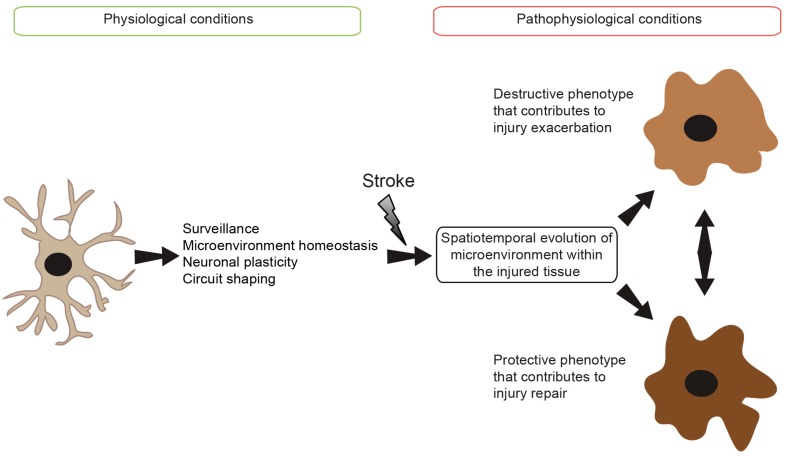
Scheme illustrating the spatiotemporal activation of microglia after stroke.

The overwhelming experimental and clinical findings outlined opposite roles of the inflammatory response in affecting ischemic stroke injury [70]. As discussed, microglial cell excessing activation is strictly controlled via the neuronal-glial crosstalk that includes CX3CL1/CX3CR1 signaling pathway. Ischemic stroke triggers activation of the microglia by impairing this crosstalk via different mechanisms [70,74]. Once activated, microglia adopt different polarized phenotypes and produce a wide range of harmful and protective inflammatory mediators [70,74]. These mediators contribute to either injury exacerbation or repair depending upon the spatiotemporal evolution of structural damage that is characterized by specific signals perceived by the microglia [74] (Figure 1). Furthermore, MDMs that are recruited to the injured tissue following BBB breakdown adopt a similar morphology to resident microglial cells [7]. However, recent findings highlighted both overlapping and many distinct functional roles of MDMs in influencing injury compared to resident microglia [7]. Here, we will summarize what is actually known about the role of resident microglia and MDMs in ischemic stroke pathobiology.

### 4.1. Resident Microglia

Microglia are rapidly activated within few minutes in the acute phase of ischemic stroke peaking [75]. Activation of the microglial cells peaks around Days 2/3 and persists for several weeks following stroke onset [76]. In the ischemic lesion core, activation of the microglia is triggered essentially by the excitotoxic signals generated during the ischemic cascade [77], whereas in the peri-infarct regions, activation of the microglia is triggered by DAMPs, which elicit a strong inflammatory response [78]. Additionally, microglia can be activated via its purinergic receptors that recognize and bind the extracellular nucleotides adenosine triphosphate (ATP) and adenosine diphosphate (ADP) that are released by dysfunctional neurons within the injured tissue [79]. Once activated, microglia change their phenotype by adopting different morphologies that are tightly associated to the spatial structural damage. Precisely, once ischemic stroke occurs the highly ramified microglia can be detected under three different distinct morphologies, a morphology characterized by enlarged cell body and short ramifications found essentially in the peri-infarct regions throughout the post-acute phases; a morphology characterized by an amoeboid cell structure with rare ramifications found as well in the peri-infarct regions throughout the post-acute phases; and a morphology characterized by a round shape representing the highly activated form of microglia found essentially nearby the core [74]. In addition to their morphological change, microglia also display an altered gene expression pattern inducing cell polarization towards functionally distinct phenotypes. Upon activation, microglia become polarized adopting different phenotypes ranging between two extremes, the classically activated M1 phenotype that is involved in pro-inflammatory actions, and the alternatively activated M2 phenotype that is involved in anti-inflammatory actions [16]. Several M2 sub-phenotypes were observed, the M2a phenotype that is involved in repair and regeneration processes, the M2b phenotype that displays immunoregulatory capacities and the acquired-deactivating M2c phenotype [80]. One general feature of all M2 phenotypes is their ability to attenuate the inflammatory response and stimulate tissue repair, whereas the M1 phenotype is known for its cytotoxic properties [80]. Both phenotypes can be distinguished by the expression of signature genes that can be used to identify those immunohistochemically [81]. It is important to specify that the M1/M2 phenotypes were initially described in macrophages [80]. However, this classification was adopted as well in microglia based on in vitro experiments where conditions are well defined, which does not necessary replicate all in vivo aspects.

The classically activated M1 microglia release a variety of pro-inflammatory factors, such as TNFα, IL1β and IL6 [82]. This exacerbates the inflammatory response and causes oxidative stress by stimulating the release of ROS as well as by excessively producing nitric oxide (NO) via inducible-nitric oxide synthase (NOS), which generates cytotoxic amounts of reactive nitrogen species (RNS) [83]. It was suggested that this phenotype induces detrimental effects on neurogenesis and aggravate long-term neurological deficits by hindering axonal regeneration [84]. It was proposed that the M1 phenotype expresses specific cell surface markers, namely CD16, CD32, FcγR and iNOS [81]. These markers are involved in many key actions associated to the M1 phenotype, namely cellular cytotoxicity, superoxide generation, degranulation and cytokine production. iNOS, which is activated by immunostimulatory cytokines is tightly associated to the M1 phenotype [80,85]. In contrast, alternatively activated M2 microglia were reported to improve brain repair and regeneration as they release several anti-inflammatory factors, such as IL4, IL10, IL13 and TGFβ that counteract the inflammatory process [82]. It was proposed that the M2 phenotype expresses specific cell surface marker, namely arginase-1 (Arg1), which was reported to have neuroprotective functions [86], as well as markers such as CD206 (mannose receptor) that binds and phagocytes mannosylated substrates and Ym1 (chitinase-like-3, Chi3l3) that prevents degradation of extracellular matrix component [82]. It has been suggested that Ym1 and CD206, which are associated to microglial M2 phenotype, were essentially found in the ischemic core contributing to tissue repair [87]. The M2 phenotype is characterized by a higher phagocytic activity compared to the M1 phenotype, thus it efficiently eliminates cell debris and promote the reconstruction of the extracellular matrix [82]. Furthermore, the M2 phenotype is characterized by the secretion of neurotrophic factors, such as insulin-like growth factor-1 (IGF1) that suppresses apoptosis and increases proliferation and differentiation of neural precursor cells (NPCs) [82], brain-derived neurotrophic factor (BDNF), TGFβ and neuronal growth factor (NGF) [88]. Elimination of the cell debris via phagocytosis and the release of anti-inflammatory cytokines by microglia translate an attempt effort to restore damaged tissue homeostasis by attenuating the detrimental effects of inflammation in order to promote regeneration and repair. Observations in experimental stroke studies showed that microglia change temporally their polarization phenotype; in the acute phase of the ischemic insult triggers the generation of M2 reparative microglia, but in the sub-acute and chronic phases microglia polarization shifts towards a M1 destructive phenotype [81]. In this regard, in vitro experiments demonstrated that ischemic neurons are able to prime microglia towards the M1 phenotype [81], whereas the chondroitin sulfate proteoglycans (CSPGs), which are components of the extracellular matrix, promote the M2 phenotype [84] These reports outline the plastic nature of microglia and the complexity of the underlying mechanisms contorting cell polarization and activity.

Recent functional experimental studies in vivo provided further insights into the complex role of microglia in ischemic stroke pathobiology [89,90]. Experimental in vivo studies demonstrating a detrimental role of microglia in ischemic stroke used essentially indirect approaches such as depletion of genes mediating signaling pathways not only in microglia but also in other brain and immune peripheral cells, such as TLR4. Ischemic damage is reduced in TLR4-depleted mice [91,92]. However, ischemic stroke increases expression of the TLR4 not only in microglia but also in astrocytes and to a lesser extent in neurons [91,93]. Additionally, TLR4 is expressed at high levels by peripheral immune cells, namely macrophages [94]. Based on these reports it might be inaccurate to conclude that the observed beneficial effects of TLR4 depletion are exclusively associated to microglial cell activity. On the other hand, recent experimental study using transgenic mice in which proliferating active microglia were selectively ablated, and not resting microglia, showed an exacerbation of the ischemic damage [95]. Importantly, the ablation of proliferating active microglia impaired the production of the anti-inflammatory cytokine IGF1 within the injured tissue [95]. Another study showed that transplantation of cultured microglia into the ischemic brain reduced ischemic damage and enhanced functional outcome [96]. Evidence suggesting that microglia are not only involved in limiting expansion of the injury, but also in stimulating tissue repair and regeneration after ischemic insult [89]. For instance, it has been shown that proliferating active microglia are present in the brain regions where adult neurogenesis occurs after ischemic stroke, namely the subventricular zone (SVZ), for several weeks. These cells seem to produce IGF1, which may contribute in supporting post-stroke neurogenesis [97]. Recent experimental study using fast in vivo two-photon intravital calcium imaging and selective microglial manipulation showed that selective elimination of the microglia leads to a significant increase in infarct size, which was reversed by microglial repopulation [98]. Microglia-mediated protection includes reduction of the excitotoxicity, as the absence of microglia disrupted calcium signaling in neurons and increased neuronal death [98]. Furthermore, spreading depolarization (SD) incidence was markedly reduced in the absence of microglia [98]. These results provide new functional in vivo insights into the role of microglia in modulating changes in neuronal network activity and SD after ischemic stroke.

### 4.2. Monocyte-Derived Macrophages (MDMs)

In contrast to microglial cell rapid response, MDMs are recruited to the injury site essentially around Days 3–7 following stroke onset [99]. In contrast to resident microglia, which were reported to be vulnerable to severe ischemic insult compromising their cell cycle progression and triggers their polarization toward the M1 phenotype, MDMs were reported to better support severe ischemic insult [100]. MDMs were shown to actively contribute to the early clearance of cell debris within the injured tissues [100]. These observations suggest that although MDMs are morphologically indistinguishable from resident microglia, they have more powerful phagocytic capacity [101], implying a more efficient role in repairing and regenerating damaged brain tissue during the post-acute phases of ischemic stroke. The emerging evidence is suggesting several functional differences MDMs depending on monocyte subsets. In human, monocytes are regrouped into three main subsets based on their CD14 and CD16 expression levels, the classical subset (CD14^++^CD16^−^), the intermediate subset (CD14^++^CD16^+^), and the non-classical subset (CD14^+^CD16^++^) [7]. In rodent, monocytes are regrouped into two main subsets based on chemokine receptor and Ly6C expression levels, the pro-inflammatory subset (CX3CR1^low^CCR2^+^Ly6C^high^) that is actively recruited to inflamed tissue contributing to the inflammatory response, and the anti-inflammatory subset (CX3CR1^high^CCR2^−^Ly6C^low^) that is continuously patrolling the lumen of vasculature contributing to the maintenance of vascular homeostasis [7]. CCR2^+^ pro-inflammatory monocytes have been shown to infiltrate the ischemic brain at the early stages differentiating into highly phagocytic macrophages [102]. Inhibition of the CCR2^+^ pro-inflammatory monocyte infiltration into the ischemic brain induced hemorrhagic transformation caused by the rupture of the injured vessels within the infarct border zone [102]. These results outline an important role of MDMs in maintaining integrity of the brain vasculature. Another study demonstrated that infiltration of the CCR2^+^ pro-inflammatory monocytes into the ischemic brain limits ischemic injury as they differentiate into M2 phenotype MDMs [103]. This study reported as well that these cells are capable of promoting polarization of the adjacent microglia towards a M2 phenotype [103]. In contrast to the role of CCR2^+^ pro-inflammatory monocytes, the role of anti-inflammatory monocytes in ischemic stroke remains unknown. However, one study showed that depletion of the anti-inflammatory monocytes did not affect brain injury [104]. In this study, the Levine/Vannucci model was used to induce stroke in adult mice. Thereby, future studies using different ischemic stroke models in adult mice are required to fully address the specific role of the anti-inflammatory monocytes in ischemic stroke pathobiology. The time course of pro-inflammatory and anti-inflammatory monocyte infiltration into the ischemic brain showed that recruitment of pro-inflammatory monocytes decreased over time, while the recruitment of anti-inflammatory monocytes significantly increased [105]. These results suggest that MDMs derived these two distinct subsets might fulfill distinct roles in ischemic stroke pathobiology.

### 4.3. Novel Sources of Microglial-Like Cells

Apart from the resident microglia and MDMs, there are few more sources from where the microglia can be found in the ischemic brain. However, some studies suggested that brain endothelial cells induce the differentiation of mature of dendritic cells (DCs) into microglia-like cells expressing high levels of CD11b, low levels of CD11c, and high levels of MHCII [106]. Whether similar mechanisms occur in the brain following ischemic stroke remains totally unknown. Finally, recent experimental findings have demonstrated that brain pericytes, which are mural cells supporting vascular functions, become activated, proliferate, leave their vascular localization and migrate towards adjacent injured tissue following ischemic stroke where they adopt an ameboid morphology and express microglial markers with phagocytic function [107,108]. These findings suggested that resident microglia might originate from ischemia-induced multipotent pericytes following ischemic insult. However, further investigations are warranted in order to fully address and elucidate this aspect.

## 5. Conclusive Summary and Therapeutic Perspectives

The major function of microglia is to maintain homeostasis and normal function of the brain. Microglia actively contribute to both ischemic stroke injury exacerbation and regeneration. This response is regulated spatiotemporally depending upon the different phases of ischemic stroke (Figure 1). The recent emerging findings outlined the extraordinary plastic nature of microglia and MDMs that adopt different functionally distinct phenotypes within the injured brain tissue. While microglia can increase the ischemic tissue damage by exacerbating the inflammatory response, these cells are required to limit injury expansion and to stimulate damaged tissue regeneration. Several experimental studies have suggested that anti-inflammatory strategies might be efficient in promoting neuroprotection in the acute phase; other studies have highlighted the crucial role of these cells in orchestrating post-stroke brain tissue repair. Activated microglia adopt functionally distinct phenotypes that elicit opposite responses ranging from exacerbating damage to reservation, making generalize anti-inflammatory strategies inappropriate for ischemic stroke therapies. The failure of clinical trials that emphasized on generally inhibiting the inflammatory response might be in part explained by the indiscriminate nature of these interventions that might have chronically inhibited the activity of microglia/MDMs leading to injury exacerbation. Understanding the complex regulatory molecular and cellular mechanisms that trigger microglial activation is crucial for the development of novel fine-tuning immunomodulating strategies that aim essentially to restore the reparative function of microglia, so that they can achieve their main task in protecting the brain. For example, counteracting the delayed post-acute shift of activated microglia from a M2 phenotype to M1 could constitute a promising therapeutic strategy to promote post-stroke regeneration. Nonetheless, to meet these challenges, it is urgent to reevaluate our understanding for the function of these cells by characterizing the mechanisms governing microglia/MDMs polarization within the injured tissue. Identifying such mechanisms is the only hope to develop fine-tuning immunomodulatory interventions that avoid the detrimental effects of total immunosuppression that can chronically deactivate all microglial subtypes. Furthermore, given the fact that MDMs contribute efficaciously to post-stroke regeneration, it may be interesting to develop special immunomodulatory interventions that specifically control the function of monocyte subsets. For example, strategies aiming at promoting the recruitment of CCR2^+^ monocyte subset and their subsequent differentiation into highly phagocytic macrophages could achieve powerful therapeutic regenerative interventions. To sum up, therapeutic strategies focusing on targeting microglia/MDMs should evolve beyond the neuroprotection in the acute phase and focus on developing novel strategies that promote repair in post-acute phases.

## Abbreviations

BBB, blood–brain barrier; rtPA, recombinant tissue-plasminogen activator; FDA, food and drug administration; ROS, reactive oxygen species; CD, clusters of differentiation; PRRs, pattern recognition receptors; TLRs, toll-like receptors; NLRs, nucleotide-binding oligomerization domain (nod)-like receptors; RLRs, retinoic acid-inducible gene-1 (RIG1)-like receptors; PAMPs, pathogen-associated molecular patterns; DAMPs, danger-associated molecular patterns; ATP, adenosine triphosphate; NF-κB, nuclear factor-kappa B; TIR, toll/interleukin-1 receptor; TRIF, TIR-domain-containing adapter-inducing interferon-β; IRF3, interferon regulatory factor-3; TNFα, tumor necrosis factor-α; TGFβ, transforming growth factor-β; TNFR1, TNF receptor 1; TNFR2, TNF receptor 2; TGFRII , TGFβ receptor type II; TGFRI, TGFβ receptor type I; NO, nitric oxide; ILRs, interleukin receptors; CX3CL1, CX3C ligand 1; CCR1, CCL1 receptor; GPCRs, G protein-coupled receptors; MAPK, mitogen activated protein kinases; TREM2, Triggering receptor expressed on myeloid cells-2; PS, Phosphatidylserine; PSRs, Phosphatidylserine receptors; BAI, brain-specific angiogenesis inhibitor-1; TIM1, T-cell immunoglobulin mucin receptor 1; MerTK, c-mer proto-oncogene tyrosine kinase; LDL, low-density lipoprotein; SR-AI, SR class AI; MARCO, macrophage receptor with collagenous structure; LOX1, lectin-like oxidized low-density lipoprotein receptor-1; FcRs, Fc receptors; CRs, complement receptors; m-CSFR, macrophage colony-stimulating factor receptor; S1R, Sigma-1 receptor; PGRMC1, progesterone receptor membrane component-1; RAGE, receptor for advanced glycation endproducts; MDMs, monocyte-derived macrophages; Iba1, Ionized calcium binding adaptor molecule-1; MHC, major histocompatibility complex; HLA-DR, human leukocyte antigen-antigen D related; ADP, adenosine diphosphate; iNOS, inducible-nitric oxide synthase; IGF1, insulin-like growth factor-1; NPCs, neural precursor cells; BDNF, brain-derived neurotrophic factor; NGF, neuronal growth factor; CSPGs, chondroitin sulfate proteoglycans; SVZ, subventricular zone; DCs, dendritic cells.
